# Virulence Biomarkers of *Bursaphelenchus xylophilus*: A Proteomic Approach

**DOI:** 10.3389/fpls.2021.822289

**Published:** 2022-02-08

**Authors:** Joana M. S. Cardoso, Sandra I. Anjo, Bruno Manadas, Hugo Silva, Isabel Abrantes, Katsunori Nakamura, Luís Fonseca

**Affiliations:** ^1^Department of Life Sciences, Centre for Functional Ecology, University of Coimbra, Coimbra, Portugal; ^2^CNC-Center for Neuroscience and Cell Biology, University of Coimbra, Coimbra, Portugal; ^3^Tohoku Research Center, Forestry and Forest Products Research Institute, Morioka, Japan

**Keywords:** pinewood nematode, pine trees, pine wilt disease, plant–nematode interactions, proteomics, secretome, virulence

## Abstract

The pinewood nematode (PWN), *Bursaphelenchus xylophilus*, one of the most serious forest pests worldwide, is considered the causal agent of the pine wilt disease (PWD). The main host species belong to the genus *Pinus*, and a variation in the susceptibility of several pine species to PWN infection is well-known. It is also recognized that there is variation in the virulence among *B. xylophilus* isolates. In the present study, we applied a quantitative mass spectrometry-based proteomics approach to perform a deep characterization of proteomic changes across two *B. xylophilus* isolates with different virulence from different hosts and geographical origins. A total of 1,456 proteins were quantified and compared in the two isolates secretomes, and a total of 2,741 proteins were quantified and compared in the nematode proteomes in pine tree extract and fungus stimuli conditions. From the proteomic analyses, a group of proteins was selected and identified as potential virulence biomarkers and shed light on putative most pathogenic proteins of this plant-parasitic nematode. Proteomic data are available *via* ProteomeXchange with identifier PXD029377.

## Introduction

The pinewood nematode (PWN), *Bursaphelenchus xylophilus*, is the causal agent of the pine wilt disease (PWD), one of the most serious forest pests in the world and responsible for high economic and ecological losses. It is vectored by insects mainly belonging to the genus *Monochamus* and has, as main host species, trees of the genus *Pinus*. Native from North America, at the beginning of the 20^th^ century, PWN was introduced in Japan, which became responsible for massive mortality of native pine trees, and then spread to neighboring East Asian countries (China, Taiwan, South Korea) during the 1980s (Futai, 2013). Probably, with a Japanese origin (Mallez et al., 2021), PWN was first reported in Europe, in continental Portugal, in 1999 (Mota et al., 1999) and spread to Spain (Abelleira et al., 2011; Robertson et al., 2011) and Madeira Island (Fonseca et al., 2012). Listed by the European and Mediterranean Plant Protection Organization (EPPO) as an A2 quarantine pest (EPPO, 2021), it represents a huge threat to forest ecosystems worldwide.

*Bursaphelenchus xylophilus* is a migratory endoparasitic nematode that is able to feed on parenchyma cells of live pine trees, migrating and spreading throughout the xylem tissues, causing cell destruction, and also to feed on fungi colonizing the dead or dying trees. During the last 20 years, advances have been made for understanding the molecular bases of these PWN-host interactions and pathogenic mechanisms. Main advances emerged from transcriptomic and genomic studies on *B. xylophilus* (Kikuchi et al., 2007, 2011; Kang et al., 2009; Figueiredo et al., 2013; Espada et al., 2016; Tsai et al., 2016), which revealed some special features of *B. xylophilus* such as the possession of cell wall-degrading enzymes, its evolutionary origin, and also constituted fundamental data that allowed the development of postgenomic studies (Shinya et al., 2013a).

Proteins are the final product of gene regulation and provide the final evidence of the function of a gene. Therefore, proteomic studies are fundamental to find out what proteins are produced and clarify which molecules are directly involved in the parasite-host interaction. From these, the secreted proteins have been of particular interest as they are directly involved in this interaction. The first complete profile of *B. xylophilus* secretome was achieved by Shinya et al. (2013b) that identified several secreted proteins potentially associated with *B. xylophilus* pathogenicity. In another study, a quantitative and comparative proteomic analysis of the secretome of *B. xylophilus* with the secretome of the closely related but non-pathogenic nematode, *B. mucronatus*, was performed, and proteins associated with peptidase activity, glycosyl hydrolase activity, and with peptidase inhibitor activity were found increased in *B. xylophilus* secretome (Cardoso et al., 2016). Additionally, the secretomes of *B. xylophilus* under the stimuli of pine species with different kinds of susceptibility to PWN were also compared, and differences detected in these secretomes highlighted diverse responses from the nematode to overcome host defenses with different susceptibilities (Silva et al., 2021).

Besides the variation in PWN host susceptibility, the nematodes themselves present a variation in their virulence level among isolates (Aikawa et al., 2003a). Several studies reported that the virulence level is usually correlated with the ability of the pathogen to multiply within the host, varies among isolates, and can be related with the geographical isolation, host trees, and environmental stresses (Kiyohara and Bolla, 1990; Aikawa et al., 2003a; Wang et al., 2005; Aikawa and Kikuchi, 2007; Shinya et al., 2012; Filipiak, 2015). Differences in gene expression patterns and genetic diversity between virulent and avirulent isolates have been reported and showed that the most significant variation between the two forms was observed in growth, reproduction, and oxidoreductase activities (Ding et al., 2016). Furthermore, some genes affected by genomic variation had potential roles in pathogenesis, such as putative effectors or digestive peptidases, which could lead avirulent isolates to display low ingestion of nutrients and provoke a delay in development (Palomares-Rius et al., 2015; Filipiak et al., 2021). Recently, in a semi-quantitative proteomic study, the comparative secretome analysis among four *B. xylophilus* isolates with different levels of virulence has been carried out and four candidate virulence determinants identified: one lipase, two cysteine peptidases, and glycoside hydrolase family 30 (Shinya et al., 2021).

In this study, a short-GeLC approach, in combination with the Sequential Window Acquisition of All Theoretical Mass Spectra (SWATH-MS) acquisition method, was used to perform a deep characterization of proteomic changes across two *B. xylophilus* isolates with different virulence and in different conditions, pine extract (PE), and fungus stimuli to discover virulence biomarkers and shed light on the most pathogenic proteins of this plant-parasitic nematode.

## Materials and Methods

### Nematode Cultures and Reproductive Ability Assays

Nematodes from the Portuguese virulent isolate BxPt17AS (BxV) originally isolated from *P. pinaster* and the avirulent Japanese isolate C14-5 (BxAv) originally isolated from the insect vector *M. alternatus* emerged from *P. densiflora* (Aikawa et al., 2003b; Aikawa and Kikuchi, 2007) were maintained in culture plates of the fungus *Botrytis cinerea* grown on Malt Extract Agar medium at 25°C. The BxPt17AS was used in previous studies as a virulent isolate (Cardoso et al., 2016, 2019, 2020; Silva et al., 2021) and the C14-5 as an avirulent isolate (Mota et al., 2006; Aikawa and Kikuchi, 2007; Filipiak, 2015).

To confirm the virulence status of each isolate, the reproductive ability in the fungus *B. cinerea* and in *P. pinaster* seedlings was determined. In the case of the fungus, 10 adult females and 10 adult males/isolate were individually separated under stereomicroscope, hand-picked, and placed in individual plates colonized with *B. cinerea* (10 plates/isolate). Twenty-one days after inoculation, nematodes were collected with sterile water (SW) from the fungus plates. The resulting nematode suspension was then sieved through a 20-μm sieve and quantified.

In the case of the *P. pinaster* seedlings, nematodes of each isolate were collected from fungus plates, used for maintenance of the isolates, and washed with SW. The resulting nematode suspension was sieved through a 20-μm sieve and used to inoculate 2-3-year-old *P. pinaster* seedlings. Approximately 3,000 nematodes (mixed developmental stages) were inoculated/seedling (5 seedlings/isolate). As control, 5 seedlings were inoculated with SW. The inoculation procedure was performed as described in Pimentel et al. (2017). Pinewood nematode inoculated and control seedlings were kept in a greenhouse at a temperature range of 20–25°C, randomly distributed, and watered two times/week. The development of the symptomatology (yellowing/browning of the leaves) was examined and registered weekly. The reproductive ability of each isolate in *P. pinaster* was assessed 32 days after inoculation. The seedlings were cut at the soil surface level, and the aerial part (main stem and branches) and roots were separated. Nematodes were extracted from the entire seedlings (aerial part and roots) using the Whitehead and Hemming tray method (Whitehead and Hemming, 1965; EPPO, 2013) and quantified.

Statistical analyses on the reproductive ability of BxPt17As and C14-5 isolates in the fungus *B. cinerea* and in *P. pinaster* seedlings were performed independently, using ANOVA (significance level *p* < 0.05). To achieve homogeneity of variance, a Log10 transformation was done. Statistical analyses were conducted using software IBM SPSS Statistics for Windows, version 28.0 (IBM Corp).

### Pine Extract Stimuli Assay

Pine extracts were prepared from 2-year-old *P. pinaster* seedlings as previously described (Cardoso et al., 2016), and the obtained solution was used as a pine stimulus. Mixed developmental nematode stages of each isolate (BxPt17As and C14-5) were collected from fungal cultures as described above and washed with SW. About 10^6^ nematodes/replicate were resuspended with 5 ml of PE previously prepared and incubated overnight at 25°C in 10-cm Petri dishes. Six replicates/stimulus were performed. Nematodes were then sedimented by centrifugation and separated from the supernatant containing the secreted proteins (± 5 ml). Samples having the secreted proteins and samples with the nematodes were stored at –80°C until the proteomic analysis of the secreted proteins (secretome) and whole nematode proteins (proteome), respectively. Nematodes from each isolate collected from fungus cultures and not exposed to PE were also stored at –80°C for analysis of the whole nematode proteins (proteome) under fungus conditions.

### Sample Preparation for Proteomic Analysis

For the preparation of secreted proteins, an internal standard [(IS) – the recombinant protein maltose-binding protein fused with green fluorescent protein (MBP-GFP)] was added in equal amounts (1 μg of recombinant protein) to each sample (Anjo et al., 2019), and the supernatants with the secreted proteins were completely dried under a vacuum using a Speedvac Concentrator Plus (Eppendorf). The resulting pellets were resuspended in an SDS-Sample buffer, aided by steps of ultrasonication (using a 750W Ultrasonic processor) and denaturation at 95°C. In addition to the individual replicates (6 in the case of the BxAv isolates and 4 replicates of the BxV), two pooled samples were created for protein identification and library creation by combining one-sixth of each replicate.

For preparation of whole nematode proteome, nematode pellets were lysed in 1-mM Tris-HCl pH 7.4 as described in Anjo et al. (2017), followed by protein precipitation using TCA-Acetone (Anjo et al., 2016). Protein pellets were resuspended as described above, and 50 μg of total protein/sample (6 replicates/condition) was used for SWATH-MS. Four pooled samples were also created for protein identification/library creation by combining 15 μg of each replicate. All samples were spiked with equal amounts of internal standard (1 μg of MBP-GFP) prior to protein digestion.

Samples were then digested as previously described (Anjo et al., 2015; Cardoso et al., 2016).

### Protein Quantification by Sequential Windowed Acquisition of All Theoretical Mass Spectra

All samples were analyzed on a NanoLC™ 425 System coupled to a Triple TOF™ 6600 mass spectrometer (Sciex^®^) using two acquisition modes: (i) the pooled samples were analyzed by information-dependent acquisition (IDA), and (ii) the individual samples by the SWATH-MS mode. The ionization source was the OptiFlow^®^ Turbo V Ion Source equipped with the SteadySpray™ Micro Electrode (1–50 μL). The chromatographic separation was carried out on a YMC-Triart C18 Capillary Column 1/32″ (12 nm, S-3 μm, 150 mm × 0.3 mm) and using a YMC-Triart C18 Capillary Guard Column (0.5 μm × 5 mm, 3 μm, 12 nm) at 50°C. The flow rate was set to 5 μl min^–1^, and mobile phases A and B were 5% DMSO plus 0.1% formic acid in water or acetonitrile, respectively. The LC program was performed as follows: 5–35% of B (0–40 min), 35–90% of B (40–41 min), 90% of B (41–45 min), 90–5% of B (45–46 min), and 5% of B (46–50 min). The ionization source was operated in the positive mode set to an ion spray voltage of 4,500 V, 10 psi for nebulizer gas 1 (GS1), 15 psi for nebulizer gas 2 (GS2), 25 psi for the curtain gas (CUR), and source temperature (TEM) at 100°C.

For IDA experiments, the mass spectrometer was set to scanning full spectra (m/z 350–1,250) for 250 ms, followed by up to 100 MS/MS scans (m/z 100–1,500) per cycle, in order to maintain a cycle time of 3.298 s. The accumulation time of each MS/MS scan was adjusted in accordance with the precursor intensity (minimum of 30 ms for a precursor above the intensity threshold of 1,000). Candidate ions with a charge state between + 1 and + 5 and counts above a minimum threshold of 10 counts/s were isolated for fragmentation, and one MS/MS spectrum was collected before adding those ions to the exclusion list for 15 s (a mass spectrometer operated by Analyst^®^ TF 1.7, Sciex). The rolling collision was used with a collision energy spread (CES) of 5. For SWATH experiments, the mass spectrometer was operated in a looped product ion mode (Gillet et al., 2012) and specifically tuned to a set of 90 overlapping windows, covering the precursor mass range of 350–1,250 m/z. A 50 ms survey scan (350–1,250 m/z) was acquired at the beginning of each cycle, and SWATH-MS/MS spectra were collected from 100 to 1,800 m/z for 35 ms, resulting in a cycle time of 3.251 s. The collision energy for each window was determined according to the calculation for a charge +2 ion centered upon the window with variable CES according to the window.

Peptide identification and library generation were accomplished by searching all the IDA samples using the ProteinPilot™ software (v5.1, ABSciex^®^) with the following parameters: (i) search against the annotated *B. xylophilus* protein database obtained from Wormbase Parasite derived from BioProject PRJEA64437 (Kikuchi et al., 2011) and MBP-GFP (IS); (ii) acrylamide alkylated cysteines as fixed modification; and (iii) trypsin as digestion type. An independent False Discovery Rate (FDR) analysis using the target-decoy approach provided with Protein Pilot software was used to assess the quality of the identifications, and positive identifications were considered when identified proteins and peptides reached 5% local FDR (Tang et al., 2008).

Quantitative data processing was conducted using SWATH™ processing plug-in for PeakView™ (v2.0.01, ABSciex^®^) (Lambert et al., 2013). After retention time adjustment using the MBP-GFP peptides, up to 15 peptides, with up to five fragments each, were chosen/protein, and quantitation was attempted for all proteins in the library file that were identified from ProteinPilot™ search. Only proteins with at least one confidence peptide (FDR < 0.01) in no less than three of the four/six replicates condition and with at least three transitions were considered. Peak areas of the target fragment ions (transitions) of the retained peptides were extracted across the experiments using an extracted-ion chromatogram (XIC) window of 3 min with 100 ppm XIC width.

The proteins’ levels were estimated by summing all the transitions from all the peptides for a given protein that met the criteria described above (an adaptation of Collins et al., 2013) and normalized to the levels of the internal standard or total intensity of each replicate, in the case of secretome or proteome samples, respectively.

For the analysis of the protein results and identification of the differentially regulated proteins, two non-parametric statistical tests were employed, considering the number of comparisons. A Mann-Whitney U test performed in InfernoRDN (version 1.1.5581.33355) (Polpitiya et al., 2008) was used in the case of the secretome samples. For the proteome analysis, a Kruskal–Wallis test was made to select the proteins, which were statistically different between all the comparisons, followed by the Dunn’s test of Multiple Comparisons, with Benjamini–Hochberg *p*-value adjustment, to determine in which comparisons statistical differences were found. All the analyses were completed using the normalized protein levels, and a *p*-value of 0.05 was defined as a cut-off.

The heatmaps from the quantified proteins were performed in Morpheus (2021), and each row corresponds to a different protein, with its normalized protein levels.

Mass spectrometry proteomics data have been deposited to the ProteomeXChangeConsorsium through the PRIDE (Perez-Riverol et al., 2019) partner repository with the data set identifier PXD029377.

### Functional Annotation

The Gene Ontology (GO) annotations were achieved with OmicsBox (2019) based on the BLAST against the non-redundant protein database NCBI and the InterPro database using the default settings in each step. The GO analysis was done in three categories: (i) molecular function, which defines molecular activities of gene products; (ii) a cellular component, which describes where gene products are active; and (iii) the biological process, which clarifies the pathways and larger processes made up of the activities of multiple gene products. Gene ontology enrichment analysis was conducted for the proteins increased in a condition against the total of quantified proteins. It was performed in OmixBox using statistical Fisher’s Exact Test associated and a *p*-value of 0.05 as a cut-off (Götz et al., 2008). Functional annotation of selected proteins was complemented with Kyoto Encyclopedia of Genes and Genomes (KEGG) pathway analysis (Kanehisa and Goto, 2000) using OmixBox.

## Results

### *Bursaphelenchus xylophilus* Isolates Reproductive Ability on Fungus and on *Pinus pinaster*

The number of nematodes, recovered from *B. cinerea* cultures of BxPt17AS isolate, was higher than the number of nematodes from C14-5 isolate. Additionally, the number of nematodes/g, extracted from the entire *P. pinaster* seedlings with C14-5 isolate, was significantly lower than from BxPt17AS-inoculated seedlings (Table 1). These results reflect the higher reproductive ability of Bx17AS (BxV) over C14-5 (BxAv) in both fungus and *P. pinaster*.

**TABLE 1:** Number of *Bursaphelenchus xylophilus* recovered from *Botrytis cinerea* cultures and from *Pinus pinaster*-inoculated seedlings.

Conditions	*Bursaphelenchus xylophilus* isolates	
	BxPt17AS (BxV)	C14-5 (BxAv)
*Botrytis cinerea*	2,656.4 ± 1042.4**^a^**	22.7 ± 12.8 **^b^**
*Pinus pinaster* seedlings	405.1 ± 239.1**^1^**	128.3 ± 48.6**^2^**

*Statistical analysis based on ANOVA for the average number of B. xylophilus (± standard deviation) recovered from one plate of fungus (different letters for p < 0.05) and per gram of pine seedling (different numbers for p < 0.05).*

### Differentially Secreted Proteins

**FIGURE 1:**
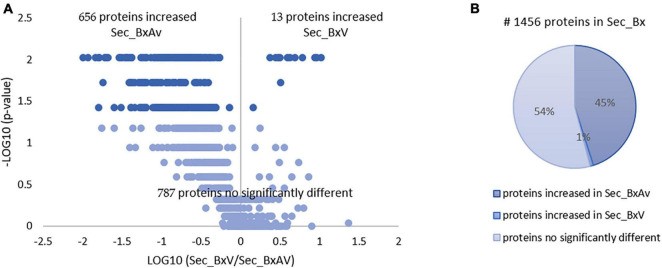
Quantitative analysis of secreted proteins of *Bursaphelenchus xylophilus* isolates. Volcano plot **(A)** and pie chart **(B)** reflecting the results from the statistical analysis of the 1,456 proteins quantified among the secretomes of *B. xylophilus* virulent (Sec_BxV) and avirulent (Sec_BxAv) isolates. Statistical analysis was performed by the Mann-Whitney U test, and statistical significance was considered for *p* < 0.05.

From the SWATH-MS analysis of the secreted proteins, a total of 1,456 proteins were quantified and compared between BxV and BxAv secretomes (Supplementary Table 1). From these, 669 were differentially expressed (*p* < 0.05), 656 increased in BxAv secretome (Sec_BxAv), and 13 in BxV secretome (Sec_BxV) (Figure 1).

From the 13 proteins found increased in BxV secretome, five were associated with peptidase activity, belonging to three groups: serine, aspartic, and cysteine peptidases. One cellulase, two proteins with lipase activity, and a venom allergen-like protein were also found increased in BxV secretome. Additionally, a γ-interferon-inducible lysosomal thiol reductase (GILT) was identified. Three of the 13 proteins could not be identified (Table 2).

**TABLE 2:** Description of increased proteins in *Bursaphelenchus xylophilus* virulent secretome (Sec_BxV) based on functional annotation.

Activity	Description	Protein ID
Peptidase	Serine peptidase	BXY_0963700.1
	Serine peptidase	BXY_1121700.1
	Serine peptidase	BXY_1703500.1
	Aspartic peptidase	BXY_0579700.1
	Cysteine peptidase	BXY_0101000.1
Glycoside hydrolase	Cellulase (GH45)	BXY_1261000.1
Hydrolase	Lipase	BXY_0707300.1
	Lipase	BXY_0824600.1
Unknown	Venomallergen-likeprotein	BXY_1378500.1
Oxidoreductase	γ-interferon-inducible lysosomal thiol reductase (GILT)	BXY_0504300.1
Putative proteins with no description		BXY_0927300.1
		BXY_0174200.1
		BXY_0073000.1

After KEGG analysis, three of these proteins were associated with five metabolic pathways: the GILT BXY_BXY_0504300.1 associated with antigen processing and the presentation pathway (Ko4612); the cellulase BXY_1261000.1 with starch and sucrose metabolism (Ko00500); and (iii) the lipase BXY_0707300.1 with lysosome (Ko04142), cholesterol metabolism (Ko04979), and steroid biosynthesis (Ko00100).

**FIGURE 2:**
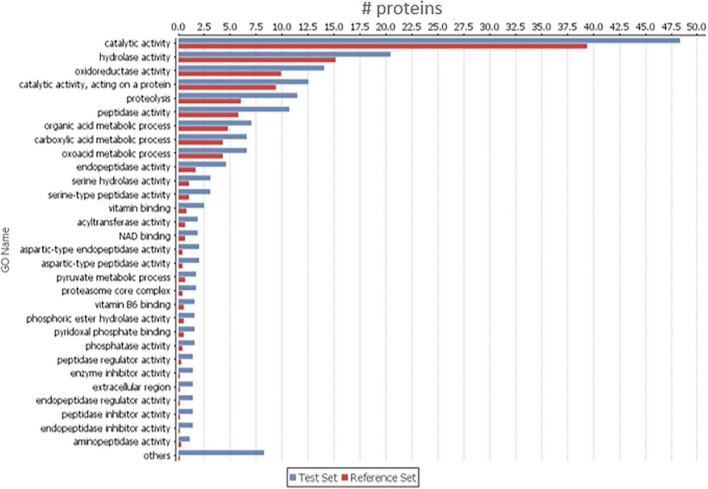
Gene ontology (GO) enrichment analysis of the 656 increased proteins in *Bursaphelenchus xylophilus* avirulent secretome (Sec_BxAv). An enriched bar chart reflecting GO enrichment analysis of the 656 proteins increased in Sec_BxAv performed against all the 1,456 quantified proteins in the secretomes using the statistical Fisher’s Exact Test with *p* < 0.05.

In order to find which group of proteins are overrepresented in the 656 increased proteins in BxAv secretome, a GO enrichment analysis was done against the 1,456 quantified proteins. This analysis revealed an enrichment of proteins associated with peptidase activity, peptidase inhibitor activity, oxidoreductase activity, and binding activity, among others (Figure 2) with GO terms associated with endopeptidase activity and endopeptidase inhibitor activity being significantly more enriched (Supplementary Table 2).

### Proteome Differentially Expressed Proteins

**FIGURE 3:**
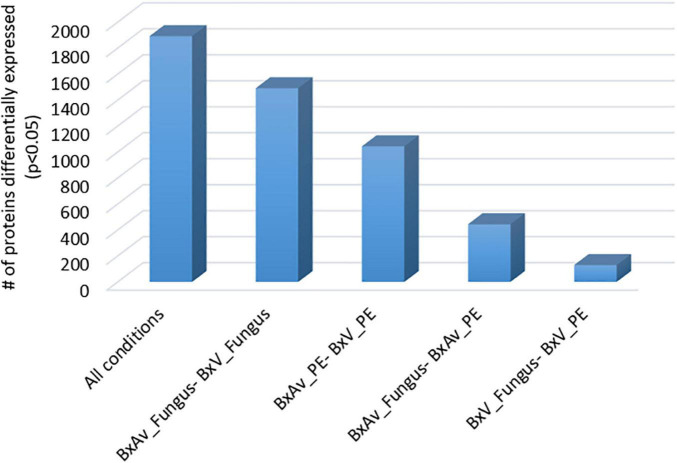
Number of differentially expressed proteins from the 2,741 proteins quantified among the proteomes of virulent *Bursaphelenchus xylophilus* isolate (BxV) and avirulent isolate (BxAv) under fungal (fungus) or under pine extract (PE) stimulus. Statistical analysis was performed by a Kruskal–Wallis test followed by the Dunn’s test of multiple comparisons, and statistical significance was considered for an adjusted *p* < 0.05.

From the SWATH-MS analysis of whole nematode proteins, a total of 2,741 proteins were quantified and compared between the nematode proteomes in different conditions: two isolates (BxV and BxAv) under fungal (fungus) or under PE stimulus (Supplementary Table 3). From these 2,741 proteins, 1,892 were differentially expressed (*p* < 0.05), considering all conditions. In pairwise comparisons, a higher number of differentially expressed protein was found between different isolates and then those of the same isolate under different condition (Figure 3).

**FIGURE 4:**
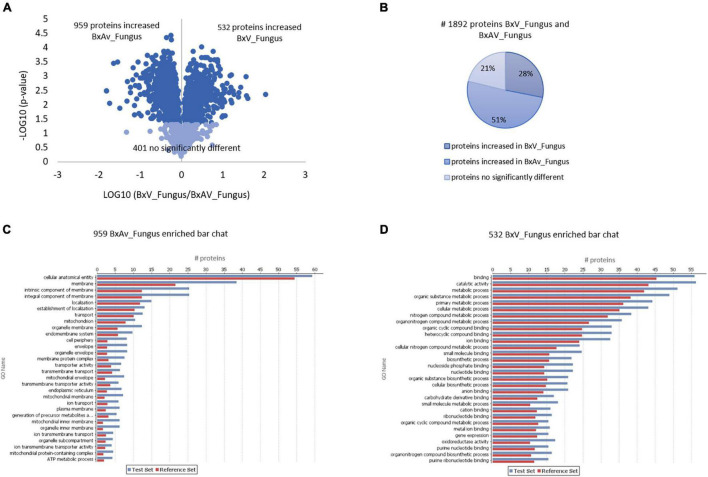
Quantitative analysis of whole nematodes proteins and gene ontology enrichment analysis of differentially expressed proteins in *Bursaphelenchus xylophilus* isolates under fungal stimulus. Volcano plot **(A)** and pie chart **(B)** reflecting the results from the statistical analysis of the proteomes of *B. xylophilus* virulent isolate (BxV_Fungus) and avirulent isolate (BxAv_Fungus) with statistical analysis of the 1,892 proteins quantified and differentially expressed (*p* < 0.05), considering all conditions, performed by the Kruskal–Wallis test, followed by the Dunn’s test for multiple comparisons. An enriched bar chart reflecting GO enrichment analysis of the 959 proteins increased in BxAv_Fungus **(C)** and 532 proteins increased in BxV_Fungus **(D)** against all the 2,741 quantified proteins in the proteomes using the statistical Fisher’s Exact Test (*p* < 0.05).

When comparing both isolates under fungal condition, a higher number of proteins (959) were found increased in BxAv_Fungus than the proteins found increased (532) in BxV_Fungus. From GO enrichment analysis, differences were noticed mainly associated with a cellular component in the increased BxAv_Fungus proteins and associated with cellular and metabolic processes and catalytic and binding activity GO terms in increased BxV_Fungus proteins (Figure 4).

**FIGURE 5:**
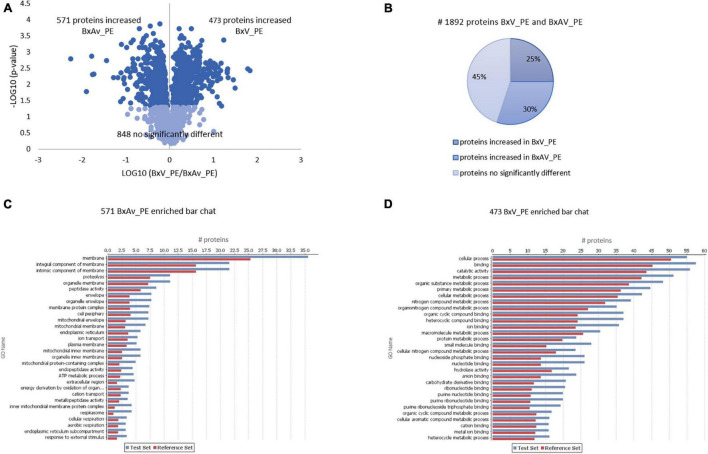
Quantitative analysis of whole nematodes proteins and gene ontology enrichment analysis of differentially expressed proteins in *Bursaphelenchus xylophilus* isolates under pine extract (PE) stimulus. Volcano plot **(A)** and pie chart **(B)** reflecting the results from the statistical analysis of the proteomes of *B. xylophilus* virulent isolate (BxV_PE) and avirulent isolate (BxAv_PE) with statistical analysis of the 1,892 proteins quantified and differentially expressed (*p* < 0.05), considering all conditions, performed by the Kruskal–Wallis test followed by the Dunn’s test for multiple comparisons. An enriched bar chart reflecting GO enrichment analysis of the 571 proteins increased in BxAv_PE **(C)** and 473 proteins increased in BxV_PE **(D)** against all the 2,741 quantified proteins in the proteomes using the statistical Fisher’s Exact Test (*p* < 0.05).

Comparing the proteomes of these two isolates under PE stimuli, 571 proteins were increased in BxAv_PE and 473 increased in BxV_PE (Figure 5). From GO enrichment analysis, an enrichment of proteins mainly associated with a cellular component in the increased BxAv_PE proteins and associated with cellular and metabolic processes and catalytic and binding activities in increased BxV_PE proteins was found (Figure 5). Overall differences in the distribution of enriched GO terms between the two isolates were similar under the two stimuli.

**FIGURE 6:**
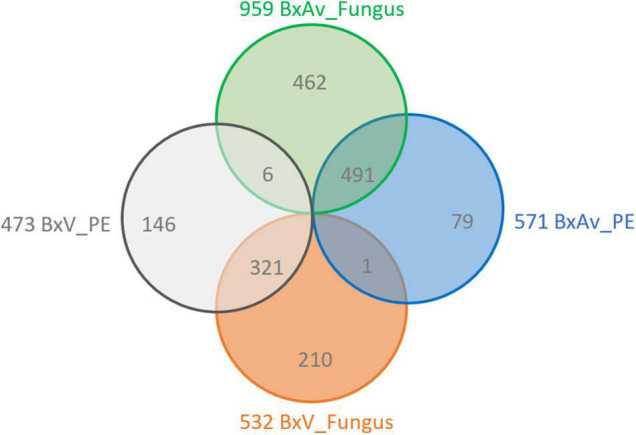
Increased proteins between each pair of conditions. A Venn diagram showing the number of unique and overlapping increased proteins between the proteomes of *Bursaphelenchus xylophilus* virulent isolate (BxV) and avirulent isolate (BxAv) under fungus (BxV_Fungus/BxAv_Fungus) and BxV and BxAv under pine extract (PE) (BxV_PE/BxAv_PE).

A comparison of increased proteins between isolates for each pair of conditions (BxV_PE/BxAv_PE and BxV_Fungus/BxAv_Fungus) revealed that most of the increased proteins of one isolate is common in both conditions, fungus, and PE (Figure 6). Moreover, the 146 proteins that were found increased in BxV proteome compared to BxAv under PE stimuli and were not increased in this isolate compared to BxAv under fungal condition were further explored as their increase could be associated with virulence status of each isolate under PE condition.

**FIGURE 7:**
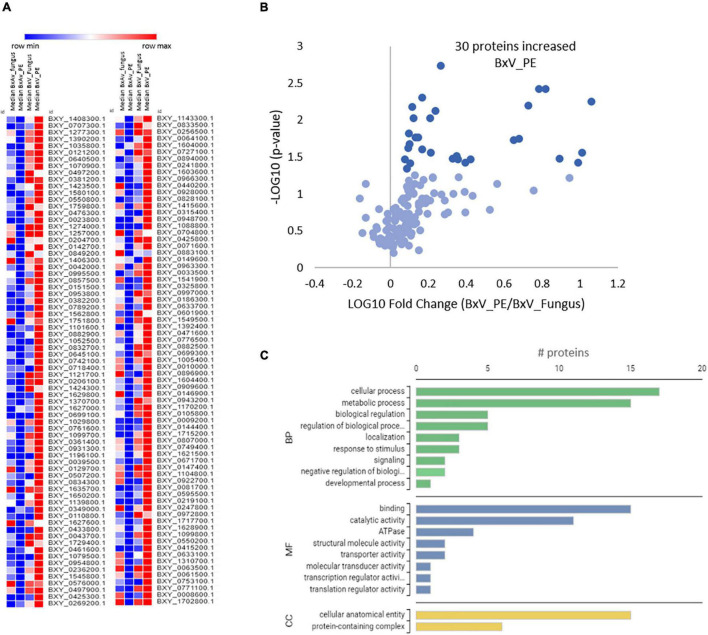
Quantitative analysis of selected 146 nematode proteins and gene ontology (GO) enrichment analysis of 30 differentially expressed proteins in *Bursaphelenchus xylophilus* isolates under pine extract (PE) stimulus. Heatmap of a normalized protein level in the proteomes of *B. xylophilus* in the four conditions: avirulent isolate under fungal stimulus (BxAv_Fungus) and under PE stimulus (BxAv_PE) and virulent isolate under fungal stimulus (BxV_Fungus) and under PE stimulus (BxV_PE) **(A)**. Volcano plot reflecting the results from the statistical analysis of the proteomes of BxV_PE and BxV_Fungus performed by the Kruskal–Wallis test followed by the Dunn’s test for multiple comparisons and with statistical significance considered for a *p* < 0.05 **(B)**. Gene Ontology distribution of the 30 selected proteins increased in virulent proteome (BxV) based on functional annotation with biological process (BP), molecular function (MF), and cellular component (CC) GO terms **(C)**.

Quantitative differences on these 146 proteins among the four conditions revealed that most of the proteins are present in higher levels in BxV_PE (Figure 7A). Thirty of these proteins are statistically increased in BxV_PE compared to BxV_Fungus condition (Figure 7B). The GO analysis of these proteins showed that they were mainly proteins associated with cellular and metabolic processes on biological process GO terms, binding and catalytic activities on molecular function GO terms and a cellular anatomical entity and protein-containing complex on cellular component GO terms (Figure 7C). From the proteins associated with catalytic activity, there were four with peptidase activity, two aspartic peptidases (BXY_1580100.1, BXY_0718400.1), and two cysteine peptidases (BXY_1052500.1, BXY_1408300.1); one protein with cellulase activity (BXY_0433800.1); and one cytochrome P450 with oxidoreductase activity (BXY_0110800.1).

After KEGG analysis, aspartic peptidase BXY_1580100.1 was found associated with autophagy (ko04140, ko04138), protein digestion and absorption (ko04974), sphingolipid signaling pathway (ko04071), lysosome (ko04142), and apoptosis (ko04210). The cysteine peptidase BXY_1052500.1 associated with plant-pathogen interaction (ko04626) and the lysosome (ko04142) and apoptosis (ko04210) pathways. The cellulase BXY_0433800.1 associated with starch and sucrose metabolism (ko00500).

## Discussion

Significant differences in the reproductive ability of BxV (Bx17AS) and BxAv (C14-5) isolates in both fungus cultures and pine seedlings were registered, indicating the higher virulence of the isolate Bx17AS, confirmed as a virulent isolate, over the isolate C14-5. However, the isolate C14-5, described as a reference avirulent isolate, was able to reproduce in *P. pinaster* seedlings. The reproductive ability of this isolate and its avirulent status were already described on the fungus *B. cinerea* and *P. thunberggi* seedlings (Mota et al., 2006; Aikawa and Kikuchi, 2007) and also on *P. sylvestris* (Filipiak, 2015), and very low or no reproduction in both fungus and pine seedlings was reported. The reproduction of C14-5 isolate in *P. pinaster* seedlings observed in this study and not reported in other hosts could be due to the conditions of the seedlings, environmental conditions, or due to the susceptibility of the host. Avirulent isolates are known to cause the death of pine seedlings when these are exposed to stresses like low light conditions (Ikeda, 1996).

Nevertheless, differences in the level of virulence of these two *B. xylophilus* isolates in *P. pinaster* were demonstrated, and how this is reflected at the proteomic level was further explored. A highly sensitive quantitative and comparative proteomic approach was applied to analyze the secretomes and proteomes of these two *B. xylophilus* isolates with different virulence and in different conditions.

From the 1,456 proteins quantified in BxV and BxAv secretomes, 787 were secreted in similar amounts by both isolates, 656 increased in BxAv secretome, and only 13 increased in BxV secretome. Interestingly, a much higher number of proteins was secreted in larger amounts by the avirulent isolate in comparison to the virulent one. Gene ontology enrichment analysis of these increased proteins in an avirulent isolate revealed that there was an enrichment of several GO categories and terms with those more significantly enriched being associated with molecular functions such as endopeptidase activity and endopeptidase inhibitor activity. A significantly more enriched number of GO categories and terms of avirulent isolates compared to virulent ones has been previously reported compared to genomes of virulent and avirulent *B. xylophilus* isolates (Filipiak et al., 2021). Also, in accordance with our results, Filipiak et al. (2021) reported that in avirulent isolates most highly represented GO terms in molecular function category were related to endopeptidases and other peptidases. Earlier studies on genomic variations among different *B. xylophilus* isolates reported that avirulent isolates share SNPs that introduce frame shift or stop codon mutations affecting protein structures and functions (Palomares-Rius et al., 2015). This same study suggested that in avirulent isolates several proteins related to proteolysis such as endopeptidases would display loss of function, and that could lead avirulent isolates to display low ingestion of nutrients and provoke a delay in development. So, the lack of function of some of these proteins in an avirulent isolate may serve as a stimulus for overexpression of that kind of proteins and consequent increased in these protein levels but, at the same time, be responsible for nematodes’ lower capacity of feeding activity and consequent lower virulence.

From the 13 proteins that were significantly increased in the secretome of the virulent isolate, five were associated with peptidase activity. Peptidases are known to be expanded in *B. xylophilus* secretome in comparison to other nematode secretomes (Shinya et al., 2013b; Cardoso et al., 2016), and 30 proteins with peptidase activity were increased in the secretome of *B. xylophilus* in comparison to *B. mucronatus*, a related but non-pathogenic nematode (Cardoso et al., 2016). In addition, five proteins also with peptidase activity were found increased in the secretome of *B. xylophilus* under *P. pinaster* stimuli in comparison to the *B. xylophilus* secretome under the stimuli of *P. pinea*, a less susceptible pine species (Silva et al., 2021). Our results support these findings, and the importance of these groups of proteins in the virulence of *B. xylophilus*, with five of the proteins increased in the secretome of the virulent isolate being associated with peptidase activity. Moreover, from the proteome analysis of both isolates in fungus and in pine extract, other four proteins with peptidase activity were found increased among the 30 proteins selected in the BxV proteome as putatively related to its virulence, two cysteine peptidases (BXY_1052500.1, BXY_1408300.1) and two aspartic peptidases (BXY_1580100.1, BXY_0718400.1). Shinya et al. (2021) reported two cysteine peptidases, Bx-CAT1 (corresponding to BXY_1052500.1) and BxCAT-2 (BXY_06188000.1), identified as potential virulent determinants and suggested their involvement in food digestion and reproductive ability. In the present study, aspartic peptidase BXY_1580100.1 was associated with several KEGG pathways, such as autophagy, protein digestion and absorption, sphingolipid signaling pathway, lysosome, and apoptosis. Autophagy is essential for feeding, fecundity, egg hatching, and survival of *B. xylophilus* under oxidative stress, contributing to its resistance to the oxidative stress induced by pine ROS metabolism, thus promoting virulence (Liu et al., 2020). As the autophagy, the programmed cell death (apoptosis) is also an important cellular mechanism in host-parasites interaction. To survive within their hosts, parasites demonstrated an ability to modulate host apoptosis pathways to their own advantage, preventing apoptosis in host cells that are inhabited by parasites and promoting apoptosis in host immune cells programmed to attack them (James and Green, 2004). The cysteine peptidase BXY_1052500.1 was reported associated with the apoptosis pathways and also with plant-pathogen interaction, lysosome, highlighting the multifunctional role of these peptidases and their potential involvement in nematode pathogenesis.

A comparative genomic study between *B. xylophilus* virulent and avirulent isolates suggested the influence of digestive proteases in the virulence of the isolates (Palomares-Rius et al., 2015). To better know the characteristics of these important proteins, a group of cysteine peptidases and aspartic peptidases secreted by *B. xylophilus* were previously characterized (Cardoso et al., 2018, 2019). From these, the cysteine peptidase BxCP11 (BXY_1052500) and the aspartic peptidase BxASP102 (BXY_0579700) were also found increased in *B. xylophilus* virulent isolate in comparison to the avirulent isolate, reinforcing their role on the pathogenicity and their potential as a possible target for the nematode control. The ability of disrupting peptidases for plant-nematode control *via* the expression of peptidase inhibitors in transgenic plants has been mentioned (Fuller et al., 2008) and should be further explored for the development of new control strategies for this important forest pathogen.

Cellulases belonging to glycoside hydrolase family 45 (GH45) degrade cellulose, one of the main constituents of the plant cell walls, and are characteristic of *B. xylophilus*, as other cellulases in plant-parasitic nematodes, such as *Meloidogyne*, *Globodera*, *Heterodera*, or *Pratylenchus*, belong to the GH5 family (Kikuchi et al., 2011; Fanelli et al., 2014). In our study, two cellulases (GH45) have been found as putative virulent biomarkers as they are increased in the secretome (BXY_1261000.1) and proteome (BXY_0433800.1) of the virulent isolate. Several *B. xylophilus* cellulases have been characterized and are suggested to have putative parasitic secretory functions that facilitate the feeding and migration processes within the pine tree (Kikuchi et al., 2004; Shibuya and Kikuchi, 2008; Cheng et al., 2010; Ma et al., 2011). The increase of these cell wall-degrading enzymes in the virulent isolate may reflect the higher capacity of this isolate to feed on plant cells and cause destruction.

Two lipases (BXY_0707300.1, BXY_0824600.1) were also found as putative virulent biomarkers as they were increased in BxV secretome. Another lipase called Bx-lip1 and corresponding to BXY_0630900.1 was identified as a potential virulent determinant by Shinya et al. (2021). Additionally, a lipase corresponding to BXY_1125700.1 was increased in *B. xylophilus* secretome under *P. pinaster* stimuli in comparison to the secretome under stimuli of *P. pinea* (Silva et al., 2021). Lipids are the main constituents of cell membranes and are known to influence pathogenesis and resistance mechanisms associated with plant-microbe interactions (Shah, 2005). Lipases can hydrolyze long-chain acyl-triglycerides into di- and monoglycerides, glycerol, and free fatty acids in many important biological processes, such as routine metabolism of dietary triglycerides to cell signaling and inflammation (Pascoal et al., 2018). The extracellular lipases of some plant pathogens such as *Fusarium graminearum* and others from the genus *Phytophthora* have been proposed as potential virulence factors (Voigt et al., 2005; Pascoal et al., 2018). In parasitic nematode species, their precise function remains to be recognized, and further functional investigations need to be implemented to investigate their ability to use the host lipids as substrate and act as virulent factors.

Another putative virulence biomarker detected in the present study was a venom allergen-like protein (BXY_1378500.1), increased in BxV secretome. These proteins are known as putative effectors involved in *B. xylophilus* migration, probably by suppressing the pine tree defense mechanism (Kang et al., 2012). A γ-interferon-inducible lysosomal thiol reductase (GILT) (BXY_0504300.1) was also found increased in the secretome of the virulent isolate and as thioredoxin-related oxidoreductase would also be associated with the detoxification against the plant defense response.

Moreover, one cytochrome P450 (BXY_0110800.1) was selected from the increased proteins in *B. xylophilus* virulent isolate proteome. These proteins are known to play important roles during the biotransformation of secondary metabolites (Urlacher and Girhard, 2012) and *B. xylophilus* can use these as a defense reaction to the secondary metabolites, such as terpenoids and cyclic aromatics, produced by the pine trees to prevent the nematode invasion (Santos et al., 2012). Some cytochrome P450 genes were overexpressed in *B. xylophilus*-infecting trees (Qiu et al., 2013) and were associated with vitality, dispersal ability, reproduction, pathogenicity, and pesticide metabolism (Xu et al., 2015).

The identification of *B. xylophilus* putative virulence proteins presented here provides a better understanding of the mechanisms that underlie the nematode infection of pine trees; however, with the lack of effective functional analysis in *B. xylophilus*, the molecular mode of action of many of these putative effectors remains to be clarified. Nevertheless, virulence biomarkers constitute key nematode targets that should be further explored for the development of new control strategies for this important forest pathogen.

## Data Availability Statement

Mass spectrometry proteomics data have been deposited to the ProteomeXChange Consortium through the PRIDE partner repository with the data set identifier PXD029377 (https://www.ebi.ac.uk/pride/archive/projects/PXD029377).

## Author Contributions

JC, SA, BM, IA, KN, and LF conceived and designed the experiments and revised and edited the manuscript. HS, SA, and JC performed the experiments and analyzed the data. JC wrote the original draft. All the authors have read and approved the final version of the manuscript.

## Conflict of Interest

The authors declare that the research was conducted in the absence of any commercial or financial relationships that could be construed as a potential conflict of interest.

## Publisher’s Note

All claims expressed in this article are solely those of the authors and do not necessarily represent those of their affiliated organizations, or those of the publisher, the editors and the reviewers. Any product that may be evaluated in this article, or claim that may be made by its manufacturer, is not guaranteed or endorsed by the publisher.
